# 

**Published:** 1872-04

**Authors:** 


☞ The JOURNAL is published Monthly, at THREE DOLLARS PER ANNUM, in advance. Each Number contains Sixty-four Pages.

### ATLANTA

### MEDICAL AND SURGICAL

### JOURNAL.

S.G.O.

EDITED BY

JOSEPH P. LOGAN, M.D.,

AND

W. F. WESTMORELAND, M.D.,

*Professor of the Principles and Practice of Surgery in Atlanta Medical College.*

X.

VOLUME [illegible].—APRIL, 1872.—NUMBER 1.

Surgeon Gen'l's Office LIBRARY Washington, D.C.

*PAX ET SCIENTIA, SED VERITAS SINE TIMORE.*

ATLANTA, GEORGIA:
PLANTATION PUBLISHING COMPANY'S PRESS.
1872.

